# Turnover of Amyloid Precursor Protein Family Members Determines Their Nuclear Signaling Capability

**DOI:** 10.1371/journal.pone.0069363

**Published:** 2013-07-18

**Authors:** Manuel T. Gersbacher, Zoë V. Goodger, Annette Trutzel, Diana Bundschuh, Roger M. Nitsch, Uwe Konietzko

**Affiliations:** Psychiatry Research, University of Zurich, Zurich, Switzerland; Tohoku University, Japan

## Abstract

The amyloid precursor protein (APP) as well as its homologues, APP-like protein 1 and 2 (APLP1 and APLP2), are cleaved by α-, β-, and γ-secretases, resulting in the release of their intracellular domains (ICDs). We have shown that the APP intracellular domain (AICD) is transported to the nucleus by Fe65 where they jointly bind the histone acetyltransferase Tip60 and localize to spherical nuclear complexes (AFT complexes), which are thought to be sites of transcription. We have now analyzed the subcellular localization and turnover of the APP family members. Similarly to AICD, the ICD of APLP2 localizes to spherical nuclear complexes together with Fe65 and Tip60. In contrast, the ICD of APLP1, despite binding to Fe65, does not translocate to the nucleus. In addition, APLP1 predominantly localizes to the plasma membrane, whereas APP and APLP2 are detected in vesicular structures. APLP1 also demonstrates a much slower turnover of the full-length protein compared to APP and APLP2. We further show that the ICDs of all APP family members are degraded by the proteasome and that the N-terminal amino acids of ICDs determine ICD degradation rate. Together, our results suggest that different nuclear signaling capabilities of APP family members are due to different rates of full-length protein processing and ICD proteasomal degradation. Our results provide evidence in support of a common nuclear signaling function for APP and APLP2 that is absent in APLP1, but suggest that APLP1 has a regulatory role in the nuclear translocation of APP family ICDs due to the sequestration of Fe65.

## Introduction

The amyloid precursor protein (APP) is a type I transmembrane glycoprotein, encoded by a single gene on chromosome 21q21, which is causally involved in Alzheimer’s disease (AD) [1]. Full-length APP is processed by a series of proteolytic cleavage reactions, mediated by the enzymes α-, β- and γ-secretase [2]. Cleavage by either α- or β-secretase results in the liberation of the soluble N-terminal fragments, sAPPα and sAPPβ, and the membrane bound C-terminal fragments, C83 and C99. The C-terminal fragments are further cleaved by γ-secretase, producing the extensively studied Aβ peptide from C99, which is regarded as a central player in AD [3]. Cleavage of APP C-terminal fragments by γ-secretase at the ε-site releases the APP intracellular domain (AICD), which has been shown to signal to the nucleus and play a role in transcriptional regulation [4], [5], [6], [7]. AICD-regulated genes include KAI1, APP, BACE1, neprilysin, and p53 [4], [8], [9], [10], [11].

APP is a member of a highly conserved family of glycoproteins, which includes the APP-like proteins 1 (APLP1) and 2 (APLP2) in vertebrates, APPL in *Drosophila,* and APL-1 in *C. elegans*. APP and APLP2 are ubiquitously expressed, with high expression in the brain [12], [13], while APLP1 expression is neuron specific [14]. Interestingly, APP-like proteins lack the Aβ domain, which is a feature unique to APP itself [15], [16]. Human *APLP2* is located on chromosome 11q23-q25 and exists in two alternatively spliced forms, one of which, similarly to APP, contains a KPI domain [15], [17], [18]. Human *APLP1* is located on chromosome 19q13.1 and, as yet, no spliced transcripts have been identified [19], [20]. All three APP family members have been shown to bind both zinc and heparin [21] and are thought to play an important role in cell adhesion in both a homo- and heterotypic manner [22], [23]. Furthermore, all family members interact with PAT1a via their basolateral sorting signal, promoting intracellular transport and increasing the processing of APP/APLPs [24].

Despite the structural homology and conserved domain structure of APP family members, it has been shown that their subcellular localization differs strikingly. A recent study by Kaden et al. showed that APP and APLP2 mainly localize within intracellular compartments, such as the ER and endosomes, with only low levels at the plasma membrane. In contrast, APLP1 was found to mainly localize to the plasma membrane, corresponding to an increased tendency to form in-trans interactions at cell-cell contacts, highlighting an important role for APLP1 in cell adhesion [25]. Further studies investigating the binding of the APP family to adaptor proteins have also identified differences between family members. For example, the binding of JIP-1 to APP has been shown to result in transcriptional activation, whereas expression of JIP-1 with APLP1 or APLP2 showed little or no transcriptional activity [26].

Studies using knockout mice have revealed important insights into the relationship between different APP family members. Single knockout for APP, APLP1, or APLP2 cause minor phenotypes due to a clear redundancy within the APP gene family [27], [28], [29]. However, it is also clear that the different family members exhibit different functions. Mice with both APP and APLP1 disrupted are viable and fertile, while those that are double knockout for both APP and APLP2 or APLP1 and APLP2 die shortly after birth [27], [28]. These findings point to an important developmental role for APLP2 that is required when either APP or APLP1 are absent. Combined APP and APLP2 double knockout exhibit a poorly formed neuromuscular junction and reduced numbers of presynaptic vesicles [30], which supports findings in Drosophila models with mutant APPL [31], implicating a role for the APP family in the regulation of synaptogenesis. Triple knockout mice, lacking all three APP family members, were found to die soon after birth and display cortical dysplasia, which resembles human type 2 lissencephaly, and is characterized by fragmented basal lamina and over-migration of neurons [32]. This phenotype is very similar to the phenotype reported in Fe65 family knockout mice [33], highlighting the importance of this AICD-binding protein in determining the functions of the APP family members.

Both APLP1 and APLP2, like APP, can be cleaved by α-, β- and γ-secretase, and intracellular domains are released after γ-secretase cleavage at the ε-site [34], [35], [36], [37]. Furthermore, BACE1 cleavage of APLP1 has recently been shown to result in the formation of an Aβ-like peptide that does not aggregate and can be a surrogate marker for increased β-cleavage in cerebrospinal fluid [38]. All three APP family members have been shown to be cleaved by caspases at their conserved VEVD motif within the C-terminus, although the significance of this cleavage is still debated [39], [40].

Proteolytic processing of APP, APLP1 and APLP2 by γ-secretase-mediated ε-cleavage generates intracellular domains (ICDs), which are stabilized by binding to the adaptor protein Fe65 and, when coexpressed with Fe65, can transactivate Gal4-Tip60 constructs [34], [41]. Transgenic mice expressing AICD and Fe65 are reported to show pathological features of AD [42]. We have previously shown that AICD is transported to the nucleus by Fe65 where, together, they bind Tip60 and form spherical nuclear complexes (AFT complexes), which localize in transcription factories [4], [43]. We have now investigated the nuclear localization of APLP1 and APLP2 ICDs upon Fe65 and Tip60 coexpression. Furthermore, we analyzed the differences in subcellular localization and turnover of the full-length proteins and their ICDs. We have identified dramatic differences in both the subcellular localization and signaling capability of APLP1 compared to the other two APP family members. Our results provide evidence for a nuclear signaling function of the APP and APLP2 ICDs and support a distinct physiological role for APLP1.

## Results

### ICDs Derived from APP and APLP2, but not APLP1, Localize to Spherical Nuclear AFT Complexes

The members of the APP family–APP, APLP1, and APLP2–show a high degree of sequence homology. In particular, the intracellular domain of APP family members is highly conserved and contains common motifs such as a caspase cleavage site and a YENPTY domain for binding of adaptor proteins. We have previously shown that AICD bound to Fe65 translocates to the nucleus and forms, together with Tip60, spherical nuclear AFT complexes [43]. We were therefore interested whether APLP1 and APLP2 intracellular domains (subsequently referred to as AL1ICD and AL2ICD) form nuclear AFT-like complexes similar to AICD.

**Figure 1:**
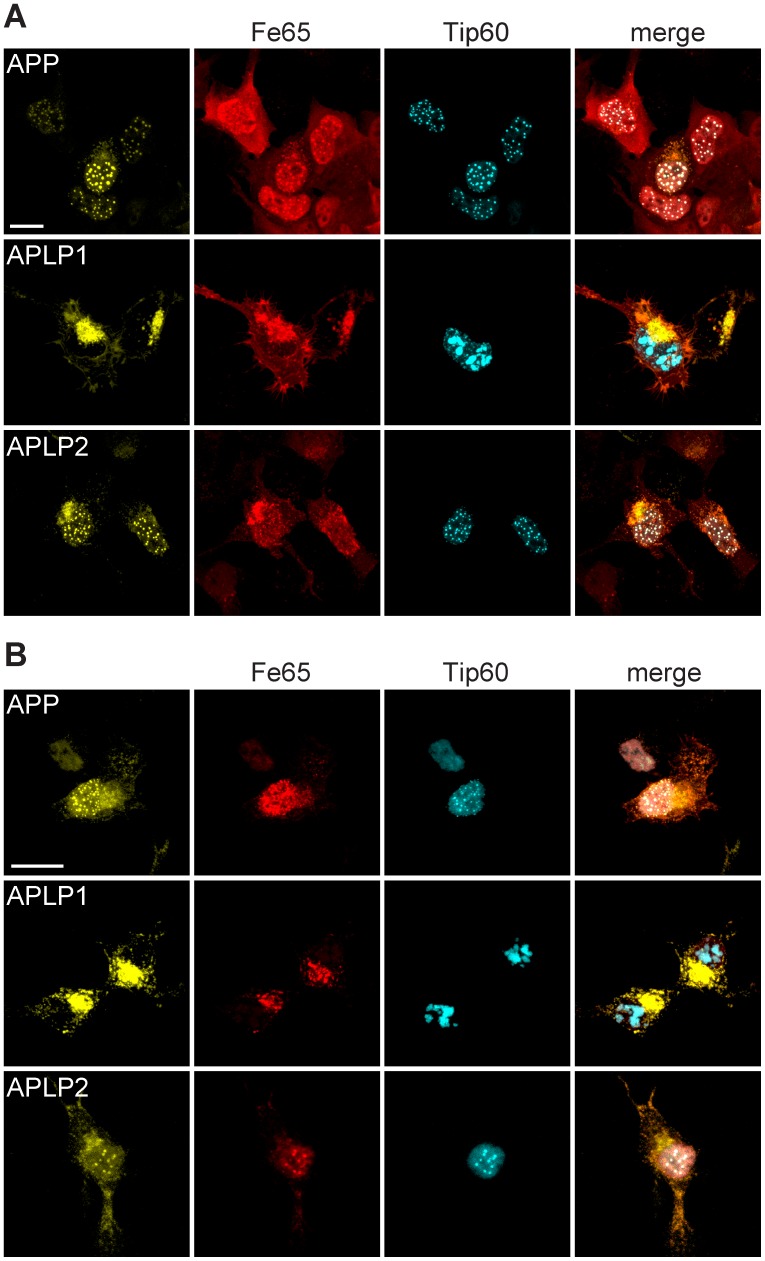
ICDs derived from APP and APLP2, but not APLP1, form nuclear AFT complexes. (A) Confocal fluorescence images of HEK cells transfected with HA-Fe65, CFP-Tip60, and APP-Cit (top row), HA-Fe65, CFP-Tip60, and APLP1-Cit (middle row), HA-Fe65, CFP-Tip60, and APLP2-Cit (bottom row). (B) Confocal fluorescence images of N2a cells transfected with HA-Fe65, CFP-Tip60, and APP-Cit (top row), HA-Fe65, CFP-Tip60, and APLP1-Cit (middle row), HA-Fe65, CTP-Tip60, and APLP2-Cit (bottom row). Note the formation of spherical AFT complexes in the nucleus of cells transfected with APP or APLP2. In contrast expression of APLP1 resulted in accumulation of Fe65 and APLP1 in extranuclear compartments and at the plasma membrane, whereas Tip60 localized to nuclear speckles. Scale bars represent 13 µm.

Coexpression of Fe65, Tip60, and APP in HEK cells resulted in the retention of Fe65-AICD complexes in the nucleus and the formation of AFT complexes as reported previously [4], [44] (Fig. 1A, top row). For detection of AFT complexes, Tip60 and APP were fused to fluorescent proteins, while Fe65 was detected by antibody staining. As already shown in an earlier publication, antibody access to AFT complexes is restricted due to their density [44]. Therefore the localization of Fe65 to AFT complexes is sometimes not as clear as observed for the fluorescently tagged proteins. Similar to AICD, AL2ICD also formed spherical AFT complexes with Fe65 and Tip60 in the nucleus (Fig. 1A, bottom row). Surprisingly, AL1ICD did not localize to the nucleus and Fe65 was retained in extranuclear compartments, colocalizing with full-length APLP1 (Fig. 1A, middle row). Cytosolic retention of Fe65 prevented the redistribution of Tip60 to nuclear spots, resulting in an accumulation of Tip60 in nuclear speckles. (Further confocal fluorescence images of HEK and N2a cells expressing APP family members together with Tip60 and Fe65 are available in Fig. S1 and Fig. S2.).

To exclude the possibility that the observed different nuclear signaling capabilities of APP family members were due to properties of the chosen cell line, experiments were repeated in N2a mouse neuroblastoma cells. Coexpression of Fe65, Tip60, and APP led to the formation of spherical nuclear AFT complexes (Fig. 1B, top row). Similar to our observations in HEK cells, AL2ICD formed AFT complexes (Fig. 1B, bottom row), while AL1ICD was not detected in nuclear structures and, due to the sequestration of Fe65 by full-length APLP1, Tip60 was localized to nuclear speckles (Fig. 1B, middle row).

### APP and APLP2 Exhibit Faster Protein Turnover than APLP1

**Figure 2:**
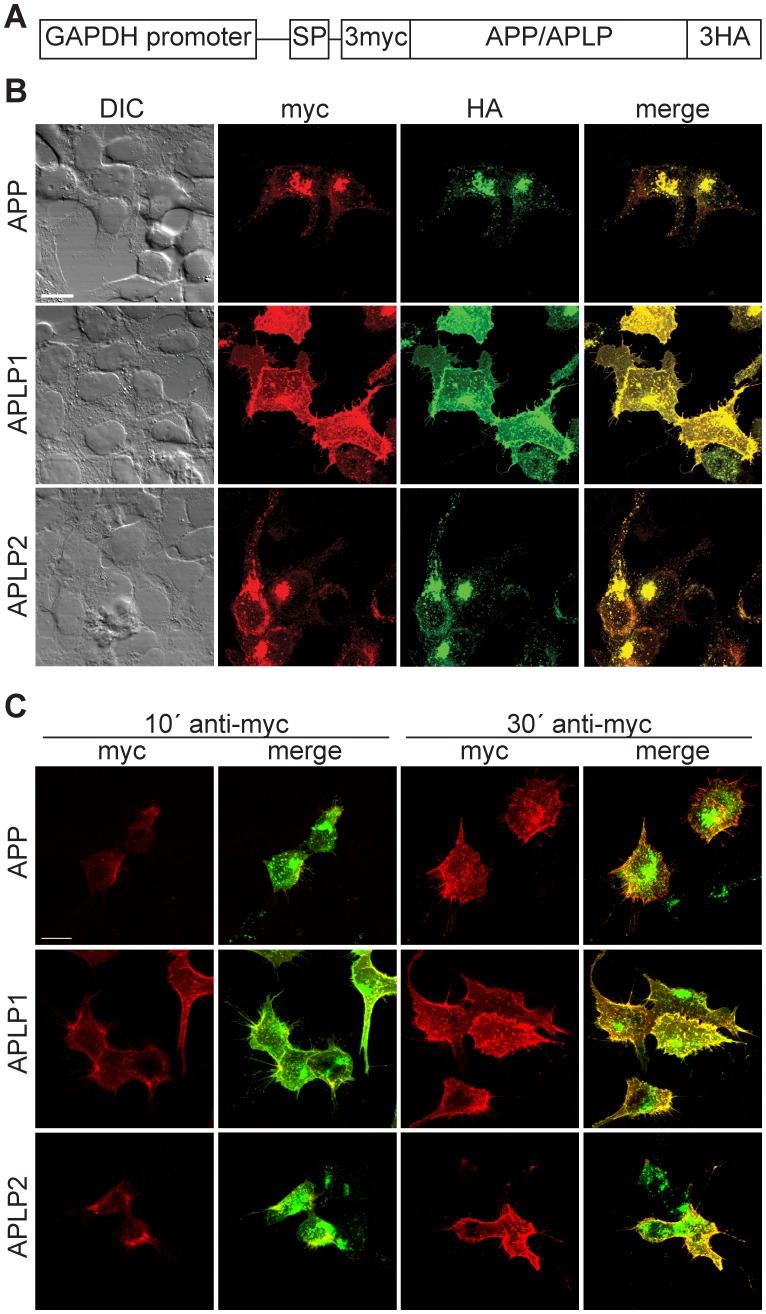
APP and APLP2 differ in their subcellular localization from APLP1. (A) Schematic presentation of APP/APLP-expression constructs with N-terminal 3 myc and C-terminal 3HA tags. The 3-myc tag is preceded by the APP signal peptide (SP) to ensure membrane insertion. (B) Confocal fluorescence images of HEK cells expressing APP (top row), APLP1 (middle row), or APLP2 (bottom row) and stained with anti-myc and anti-HA antibodies. APP and APLP2 showed a more prominent intracellular localization to vesicular structures, whereas APLP1 mostly localized to the cell membrane. (C) Confocal fluorescence images of HEK cells expressing APP (top row), APLP1 (middle row) or APLP2 (bottom row) after live antibody incubation. Incubation of cells with anti-myc antibody at 4°C for 10 minutes results in surface labeling, especially of APLP1. After 30 minutes of anti-myc antibody incubation, cell surface signals of APP and APLP2 reached a similar strength as APLP1. Cells were counter-stained with anti HA antibody after fixation. Scale bars represent 13 µm.

To further study the subcellular localization of the APP family members we expressed APP/APLP with N-terminal 3 myc tags, preceded by the APP signal peptide to ensure membrane insertion, and C-terminal 3HA tags (Fig. 2A). A GAPDH promoter was chosen for expression because the GAPDH gene is constitutively expressed at high levels in almost all tissues and expression of transgenes via this mammalian promoter yields good expression that is weaker than with viral promoters. Expression of APP family members in HEK cells showed a clear intracellular localization to vesicular structures for APP and APLP2, whereas APLP1 was mostly localized to the cell membrane (Fig. 2B). The different subcellular localization of APP and APLP1 was also observed with coexpression of Citrine-labeled APP and Cerulean-labeled APLP1 constructs (Fig. S3A). Furthermore, fluorescent resonance energy transfer (FRET) could be observed between APP and APLP2 but not between APP and APLP1 in HEK cells, primary astrocytes, and neurons (Fig. S3 B–D). Thus, in addition to the colocalization of APP and APLP2, these two family members can form heterodimers.

The N-terminal 3 myc tag-constructs allowed cell-surface labeling of APP/APLPs on living cells (Fig. 2C). Antibody incubations were performed at 4°C to inhibit endocytosis that is dependent on GTPase function. In contrast, exocytosis, which is promoted by zippering of SNARE proteins mediating vesicle fusion, is still able to occur in the absence of enzymatic activity. After 10 minutes of anti-myc antibody incubation we detected surface labeling for all three APP family members. The strongest staining was observed for APLP1. However, after 30 minutes incubation, APP and APLP2 surface staining reached similar levels as APLP1. This points towards a higher turnover of APP and APLP2 compared to APLP1 at the plasma membrane.

**Figure 3:**
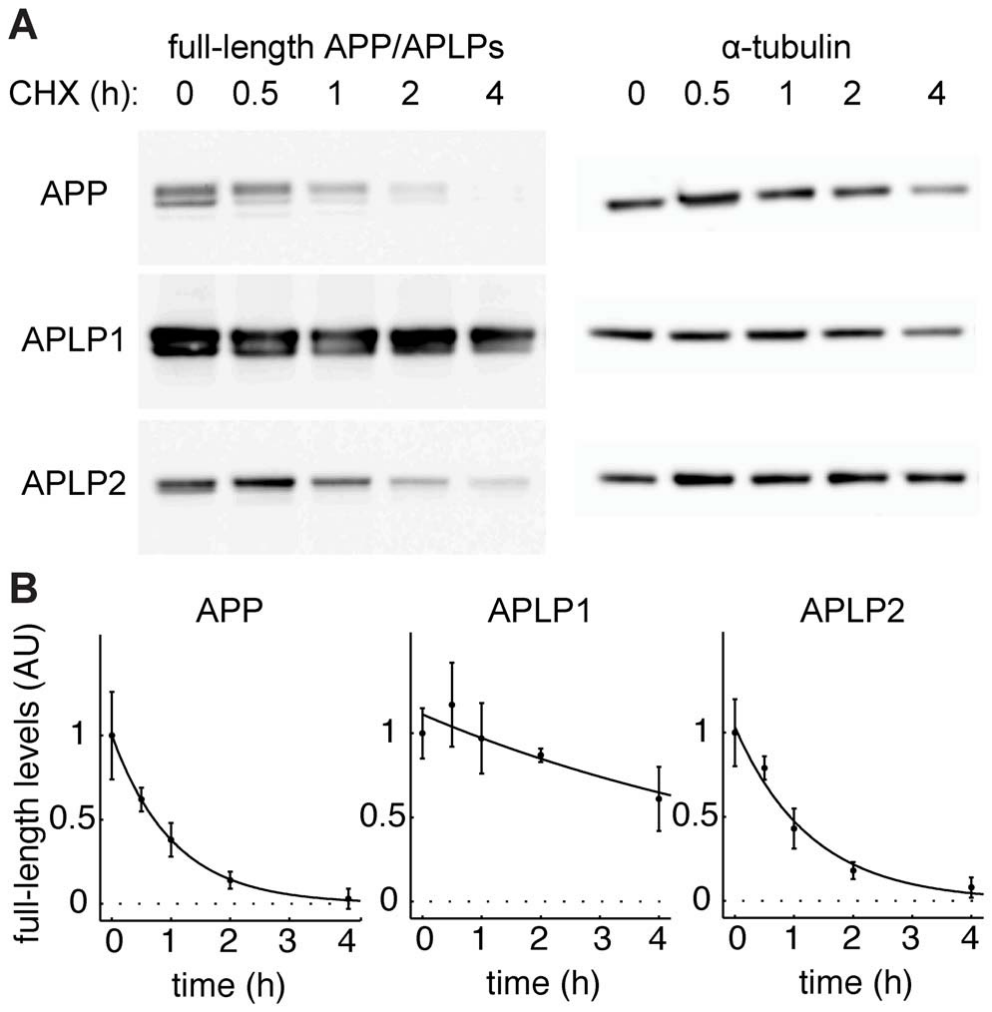
APP and APLP2 have a higher protein turnover than APLP1. (A) Western blot analysis of HEK cells transfected with C-terminally HA-tagged APP/APLPs after indicated times of protein synthesis inhibition with cycloheximide (CHX). Western blots were probed with anti-HA antibody. Note the strong accumulation of APLP1 as compared to APP and APLP2. (B) APP/APLP full-length levels from A were normalized to α-tubulin. Mean ± SEM of n = 3 are shown for each time point. Data was fitted to exponential functions by the least square approach. R^2^_(APP)_ = 0.99; R^2^_(APLP1)_ = 0.82; R^2^_(APLP2)_ = 0.98.

To measure the half-lives of the APP family members we inhibited protein synthesis for different durations. HEK cells were transfected with C-terminally 3HA-tagged APP/APLP constructs, all driven by a GAPDH promoter to ensure comparable expression levels. 24 hours after transfection, protein synthesis was inhibited with cycloheximide (CHX). Cells were harvested after the indicated time of cycloheximide incubation and levels of full-length protein determined by Western blot (Fig. 3A). APP/APLP full-length levels were normalized to the highly stable α-tubulin. APP and APLP2 displayed short half-lives of 43 minutes and 53 minutes respectively, while half-life of APLP1 was higher than five hours (308 minutes) (Fig. 3B). The much higher stability of APLP1 is also evident in the left column of figure 3A: despite the expression of all three family members under the same promoter, the levels of APLP1 were much higher, again highlighting its slower turnover.

### The N-terminus of ICDs Determines their Nuclear Signaling Capabilities

**Figure 4:**
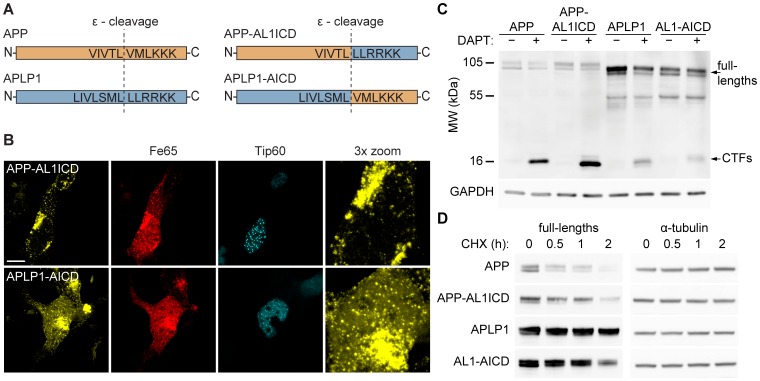
Nuclear signaling capability of APP family members is mediated by the intracellular domain. (A) Schematic representation of wildtype and chimeric APP/APLP1 constructs. (B) Confocal fluorescence pictures of HEK cells cotransfected with HA-Fe65, CFP-Tip60 and the chimeric constructs APP-AL1ICD-Cit (top row) or APLP1-AICD-Cit (bottom row). Note that AFT complexes are formed in cells expressing APLP1-AICD but not APP-AL1ICD. Scale bar represents 13 µm. (C) Western blot analysis of HEK cells transfected with wildtype or chimeric APP/APLP1 constructs after 24-hour treatment with the γ-secretase inhibitor DAPT. Western blots were probed with anti-HA antibody and accumulation of CTFs was observed with all constructs. (D) Western blot analysis of HEK cells transfected with C-terminally HA-tagged APP/APLP after indicated times of protein synthesis inhibition with cycloheximide (CHX).

To investigate whether differences in nuclear localization capability of AICD and AL1ICD are mediated by properties of their extracellular or intracellular domains, we constructed chimeric APP/APLP1 expression plasmids. ICDs of APP and APLP1 were joined at the ε-cleavage site of APLP1 and APP, resulting in the chimeric constructs APLP1-AICD and APP-AL1ICD, respectively (Fig. 4A). Chimeric constructs were cotransfected with Fe65 and Tip60 in HEK cells and AFT complex formation was observed by confocal microscopy. APP-AL1ICD did not form AFT complexes, despite preserved binding of Fe65 (Fig. 4B, top row). In contrast, nuclear AFT complex formation was observed in cells transfected with APLP1-AICD. These results suggest that the formation of AFT complexes is determined by the properties of the intracellular domain. Interestingly, the size of AFT complexes formed from APLP1-AICD appeared to be decreased when compared to AFT complexes formed from wildtype APP (Fig. 1A, top tow).

Because the chimeric proteins carry a chimeric ε-cleavage site it is possible that absent nuclear signaling of ICDs is due to impaired γ-secretase cleavage. To investigate this possibility, HEK cells were transfected with APP, APLP1 or the chimeric APP/APLP1 proteins and treated with the γ-secretase inhibitor DAPT (Fig. 4C). DAPT treatment resulted in an accumulation of C-terminal fragments (CTFs) when compared to non-treated cells, demonstrating that γ-secretase cleavage is not impaired in the chimeric proteins. In line with the previous experiments using cycloheximide, increased full-length levels and decreased CTF levels of APLP1 indicate the slower turnover of APLP1 compared to APP. Furthermore, these results suggest that the extracellular and/or transmembrane regions mediate the differences in protein turnover. To better understand the turnover of APP family members, wildtype and chimeric APP-transfected HEK cells were treated with cycloheximide to monitor the turnover of full-length proteins. As described earlier, APP turnover is much faster than APLP1 turnover (Fig. 4D). Turnover of the chimeric proteins was in between that of the wildtype proteins but resembled more closely the protein carrying the respective extracellular domain. Together, these results suggest that both extracellular and intracellular regions determine the turnover of APP family members. Moreover, it is likely that the decreased AFT complex formation for APLP1-AICD as compared to APP is due to the decreased turnover of APLP1-AICD, which would also lead to decreased AICD levels.

**Figure 5:**
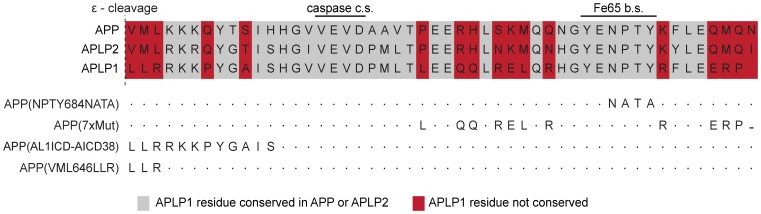
APP family members show high sequence homology. Schematic presentation of APP family ICD sequences and APP/APLP1 mutations. c.s.: cleavage site; b.s.: binding site.

**Figure 6:**
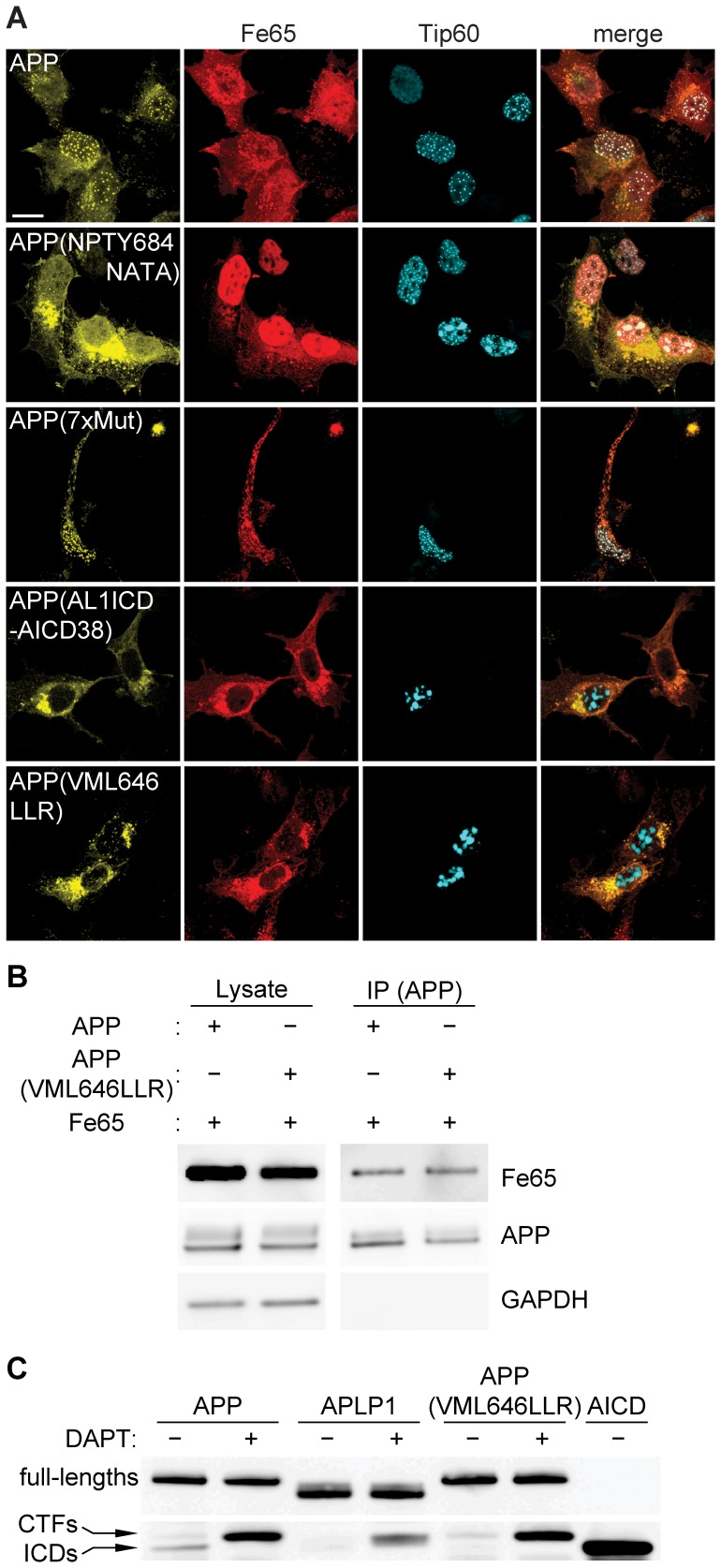
N-terminal residues of APP family ICDs are crucial for nuclear signaling capability. (A) Confocal fluorescence images of HEK cells transfected with HA-Fe65, CFP-Tip60 and cotransfected with APP-Cit or the indicated APP-Cit mutation constructs. Scale bar represents 13 µm. (B) Co-immunoprecipitation of SBP-tagged APP or APP(VML646LLR) together with HA-tagged Fe65 using Dynabeads. (C) Western blot analysis of HEK cells transfected with APP-Cit, APLP1-Cit or APP(VML646LLR)-Cit constructs after 24 hour treatment with the γ-secretase inhibitor DAPT. AICD-Cit transfected cell lysate was loaded to identify ICD bands and the membrane was probed with anti-GFP antibody.

Our results show that nuclear signaling by the APP family members is determined by properties of the ICDs, which are highly conserved and share common motifs, such as a caspase cleavage site and the YENPTY motif (Fig. 5). We reasoned that the differences in AFT complex formation capability between APP/APLP2 and APLP1 must derive from single amino acid or motif differences between the proteins. We identified 17 conserved residues in AICD and AL2ICD that are not conserved in AL1ICD and could thus be responsible for nuclear signaling (Fig. 5). To test this hypothesis, we exchanged individual or multiple residues of APP by the corresponding residues of APLP1 and investigated AFT complex formation. Mutation of the NPTY motif to NATA abolished Fe65 binding as previously reported [6] and prevented the nuclear translocation of AICD (Fig. 6A, row 2). We subsequently mutated amino acids sequentially, either alone or in combination. (See Fig. S4 for a diagram depicting all mutations and Fig. S5 for corresponding confocal fluorescence pictures.) Changing all seven non-conserved residues in the C-terminal region of AICD that is reported to bind to Fe65 [45] did not disrupt nuclear signaling (Fig. 6A, row 3; APP(7 xmut)). In contrast, when we inserted the first 12 amino acids of AL1ICD into the APP sequence (APP(AL1ICD-AICD38)), nuclear signaling was completely abolished (Fig. 6A, row 4). Further site-directed mutagenesis were done to identify the minimal set of amino acids that prevent nuclear localization of AICD. By this means we discovered that exchange of the N-terminal residues VML to the corresponding APLP1 residues LLR (APP(VML646LLR)) is sufficient to ablate AFT complex formation (Fig. 6A, row 5).

Co-immunoprecipitation of streptavidin-binding protein (SBP)-tagged APP and APP(VML646LLR) showed that Fe65 binding is not impaired by exchange of N-terminal ICD residues (Fig. 6B). Since DAPT treatment of cultures transfected with APP(VML646LLR) resulted in an accumulation of CTFs, we can also exclude the possibility that the absence of nuclear AFT complex formation for APP(VML646LLR) is due to disturbed γ-secretase cleavage (Fig. 6C). Interestingly, in DMSO control treated cells, ICDs derived from APP were detected but not from APP(VML646LLR) or APLP1.

### Proteasomal Degradation Differs between AL1ICD and AICD

**Figure 7:**
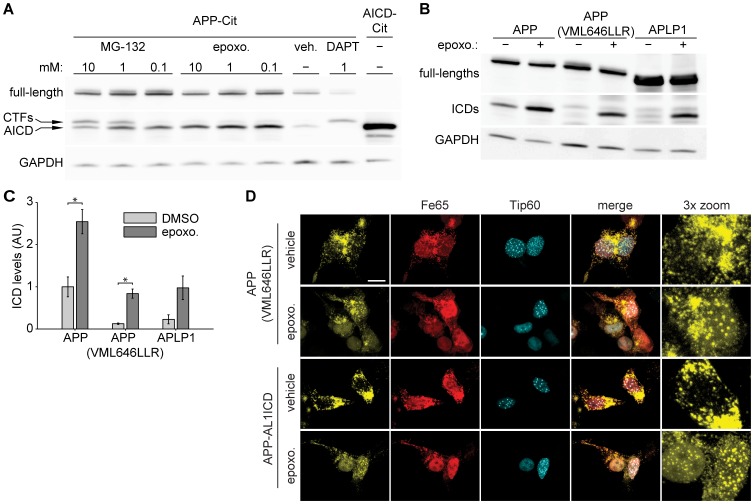
Nuclear localization of APP family ICDs is regulated by different proteasomal degradation rates. (A) Western blot analysis of HEK cells transfected with APP-Cit followed by 6 h treatment with indicated concentration of MG-132 or epoxomicin. APP-Cit transfected HEK cells treated with DMSO or DAPT and AICD-Cit transfected cells were loaded to identify CTFs and AICD bands. Membranes were probed with anti-GFP antibody. Note that MG-132 inhibits the proteasome and at higher concentrations also γ-secretase, whereas epoxomicin is a specific proteasome inhibitor. (B) Western blot analysis of HEK cells transfected with APP-Cit, APLP1-Cit or APP(VML646LLR)-Cit constructs followed by 6 hours of proteasome inhibition with epoxomicin or DMSO control treatment. The membrane was probed with anti-GFP antibody and GAPDH was used as a loading control. In the absence of proteasome inhibition ICDs generated from APP are clearly visible. (C) Quantification of ICD levels from B. Mean ± SEM of n = 3 are shown (p<0.05, t-test). (D) Confocal fluorescence images of HEK cells transfected with HA-Fe65, CFP-Tip60 and cotransfected with APP(VML646LLR)-Cit (upper rows) or chimeric APP-AL1ICD-Cit (bottom rows) mutation constructs with 6 hours epoxomicin or DMSO control treatment. Note that AFT complexes are formed after epoxomicin treatment. Scale bar represents 13 µm.

Several reports have demonstrated that AICD is degraded by the proteasome [46], [47], but little is known about AL1ICD and AL2ICD degradation. We hypothesized that APP family ICDs have a different protein turnover, resulting in different capabilities to form nuclear AFT complexes. To confirm proteasomal degradation of AICD, we treated HEK cells transfected with APP-Cit with two previously described proteasome inhibitors, epoxomicin and MG-132. Treatment with epoxomicin strongly increased AICD levels, confirming proteasomal degradation of AICD, while treatment with MG-132 resulted in an increase in CTF and AICD levels, suggesting that MG-132 inhibits the proteasome as well as γ-secretase when used in higher concentrations, as reported previously [48] (Fig. 7A). To avoid interference with secretase processing of APP, epoxomicin was used for proteasomal inhibition in the following experiments. HEK cells were transfected with APP-Cit, APP(VML646LLR)-Cit, or APLP1-Cit and treated with epoxomicin for six hours. In control treated cells, only ICDs generated from APP were detectable, while epoxomicin increased the ICDs to detectable levels for the different constructs (Fig. 7 B–C). These results show that the half-life differs between APP family ICDs and suggest that proteasomal degradation efficacy is dependent on the N-terminus generated after γ-secretase cleavage.

**Figure 8:**
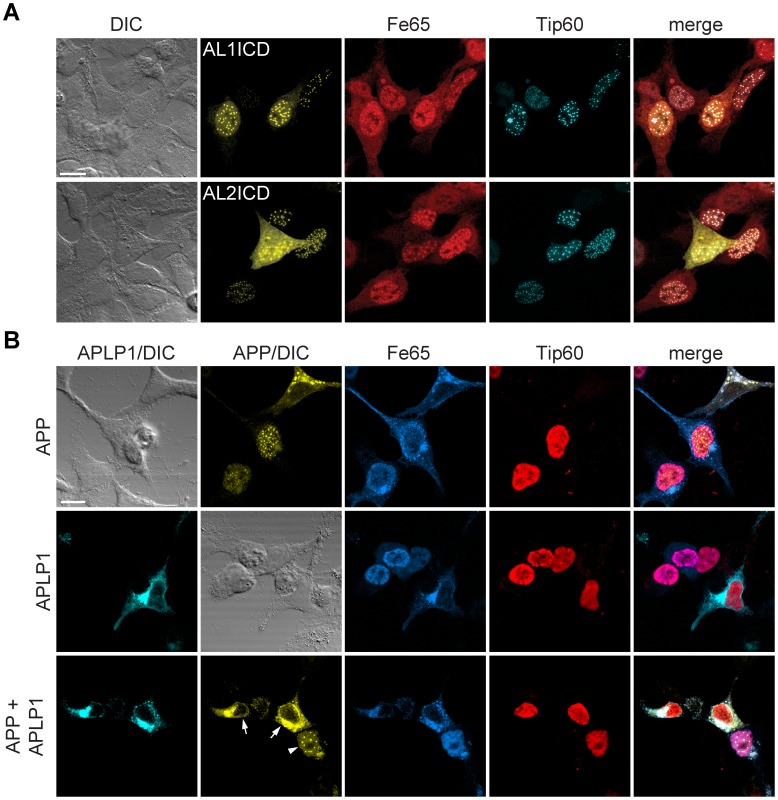
APLP1 expression prevents localization of AICD to AFT complexes. (A) Confocal fluorescence images of HEK cells transfected with HA-Fe65, CFP-Tip60 and Cit-AL1ICD (top row) or Cit-AL2ICD (bottom row). Note the nuclear localization of Al1ICD to nuclear complexes. (B) Confocal fluorescence images of HEK cells transfected with HA-Fe65, Myc-Tip60 and cotransfected with APP-Cit (top row) APLP1-Cer (middle row) or both (bottom row). Note that AFT complex formation (arrowhead) was ablated in cells expressing APP as well as APLP1 (arrows). Scale bars represent 13 µm.

Next, we asked whether the localization of APP family ICDs that do not form AFT complexes is changed after epoxomicin treatment. Both APP(VML646LLR)-Cit and APP-AL1ICD-Cit, coexpressed with Fe65 and Tip60, formed AFT complexes when treated with epoxomicin (Fig. 7D). These results show that both AICD and AL1ICD can be transferred to the nucleus and form AFT complexes but under normal conditions AL1ICD is degraded very quickly by the proteasome due to the identity of its N-terminal residue. In line with this, we showed that AL1ICD with an N-terminal fusion of Citrine that prolongs the half-life, resulted in nuclear localization of AL1ICD to AFT-like complexes when coexpressed with Fe65 and Tip60 (Fig. 8A).

### APLP1 Regulates APP Nuclear Signaling

APLP1 does not signal to the nucleus but it nevertheless binds to Fe65. We therefore investigated the influence of APLP1 expression on the nuclear signaling of AICD. We used a myc-tagged Tip60 to be able to coexpress Cerulean and Citrine-tagged APLP1 and APP. AICD cleaved from APP again translocated to nuclear AFT complexes in cells coexpressing Tip60 and Fe65 (Fig. 8B). APLP1 bound to Fe65 with higher affinity than Tip60, as seen by the relocalization of Fe65 away from Tip60 in the nucleus. Consequently, coexpression of APLP1 together with APP, Fe65 and Tip60, prevented the formation of nuclear AFT complexes that are clearly seen in cells not expressing APLP1 (Fig. 8B). These results suggest that although APLP1 does not directly signal to the nucleus, it influences AICD nuclear signaling via the sequestration of Fe65.

## Discussion

Although the APP family members share a high sequence homology and undergo similar processing, different properties and functions of the three proteins have been described [25]. Here, we provide further experimental evidence for a distinct function of APLP1 among APP family members, by reporting a unique nuclear signaling capability for the ICDs of APP and APLP2, but not APLP1.

We show that the ICDs released from APP and APLP2 localize together with Fe65 and Tip60 to spherical nuclear complexes. In contrast, the ICD released from APLP1 does not localize to the nucleus, although it is able to bind Fe65. We demonstrate that nuclear localization of AL1ICD is prevented at two steps of APLP1 processing. Firstly, slower turnover of full-length APLP1 compared to APP results in lower levels of CTFs, which are the direct precursors of ICDs. Secondly, AL1ICD undergoes faster proteasomal degradation compared to AICD. Both steps ultimately result in lower levels of AL1ICD. Our results also indicate that, although APLP1 does not signal directly to the nucleus, it has a regulatory function in AICD nuclear localization.

The expression of full-length APP or APLP2, together with Fe65 and Tip60, results in the translocation of their ICDs to nuclear AFT complexes, which is dependent on cleavage by γ-secretase [4]. In contrast, the ICD derived from APLP1 does not translocate to the nucleus. APLP1 binds and sequesters Fe65 outside of the nucleus, while Tip60 remains localized in nuclear speckles. These speckles represent a different nuclear compartment to that occupied by nuclear AFT complexes, which themselves are thought to correspond to sites of transcription [43]. Of note, AICD and AL2ICD occupy the same nuclear sites as the transcriptional activator NICD and interactions of AICD and NICD nuclear signaling have been described [49] (data not shown). This could indicate that similar genes are regulated by the ICDs of APP and APLP2. In fact, the analysis of candidate genes regulated by AICD has shown that neprilysin expression and activity is reduced in fibroblasts derived from APP or APLP2 knockout mice and is dramatically diminished in cells from double knockout mice [9]. Likewise, for genes suppressed by AICD, as reported for LRP1, the expression levels were even stronger in APP/APLP2-deficient cells compared to single APP knockout cells [50]. Taking this combined evidence into account, these results point towards an exclusive function of APP and APLP2 in transcriptional regulation.

Using cell surface labeling of living cells and determination of protein half-life time after cycloheximide-induced inhibition of translation, we clearly show that full-length APP and APLP2 have a much faster turnover than APLP1. The stability of APLP1 is evident in different experimental settings and in stark contrast to the rapid turnover of APP and APLP2, whose rapid turnover kinetics have also been reported *in vivo* [51]. Investigation of the nuclear signaling capabilities of chimeric APP/APLP1 proteins suggested that nuclear signaling capability is an intrinsic property of the ICD sequence that is present in APP but not in APLP1. We aligned the ICD sequences and identified 17 amino acids that are conserved between APP and APLP2 but differ in APLP1. To identify sequence determinants for nuclear signaling capability we individually or in combination exchanged all amino acids of AICD with the corresponding APLP1 residues. Out of all single amino acid and motif changes, only the exchange of the N-terminal amino acids VML to LLR ablated nuclear signaling of AICD. APP(VML646LLR) binding to Fe65 and processing by the secretases were intact, but the resulting ICD levels were much lower than for the APP-derived ICDs, suggesting different degradation rates.

It has been reported that AICD is degraded by insulin-degrading enzyme (IDE), the lysosomal pathway, and the proteasome, whereas degradation of ALICDs has only been poorly investigated [47], [52]. Here, we show that the ICDs of both APP and APLP1 are degraded by the proteasome. Proteasomal degradation of AL1ICD is faster than for AICD. Inhibition of proteasomal degradation by epoxomicin and the concomitant increase in ICD levels resulted in the translocation of ICDs derived from APP(VML646LLR) and the chimeric construct APP-ALICD to the nucleus. Under normal conditions the ICDs of these constructs are degraded and do not reach the nucleus. However, ICDs released from APLP1, even with epoxomicin treatment, did not translocate to the nucleus. This is probably due to the fact that APLP1, because of its slower turnover, accumulates to higher levels that bind and sequester Fe65, making it unavailable for the transport of AL1ICD to the nucleus. These results suggest that: (a) nuclear localization is an intrinsic property of all APP family ICDs, (b) a certain threshold level for ICDs has to be reached for nuclear translocation to occur, and (c) slow turnover of full-length APLP1 sequesters Fe65, rendering it unavailable for nuclear translocation of ICDs.

Since the levels and proteasomal degradation of ICDs of APP(VML646LLR) and APLP1 behave in a similar way, our results suggest that the proteasomal degradation rate of ICDs is dependent on their N-terminal residues. Interestingly, work by Alexander Varshavsky and colleagues has revealed a proteasomal pathway in which the degradation of a protein is related to the identity of the protein’s N-terminal residues [53]. According to this N-end rule, leucine, the N-terminal residue of APLP1, is a destabilizing residue, whereas valine and methionine, the N-terminal residues of APP, are stabilizing. Since both, APP and APLP1 are degraded by the proteasome and carry multiple lysines that could potentially be ubiquitinated, it is possible that APP family ICDs, as suggested previously [54], are substrates of the N-end rule pathway.

Walsh et al. reported higher stability for AL1ICD than AICD and AL2ICD when ICDs were expressed as soluble forms [37]. Similar to Walsh et al., we observe nuclear signaling for ICDs of all three APP family members when ICDs are expressed as soluble proteins (Fig. 8A). The apparent differences in localization for AL1ICD derived from full length APLP1 and soluble expressed AL1ICD are not surprising since much higher levels of ICDs are obtained from soluble expressed AL1ICD. Furthermore, we have shown that AL1ICD generated by γ-secretase cleavage carries N-terminal residues that influence degradation rate. However, the expression of soluble ICDs necessitates the addition of a methionine at the N-termini that is likely to neutralize the different degradation times of AICD and AL1ICD reported here.

Our results help to explain the profound effects seen in knockout mouse models of the APP family. Animals with a double deficiency in both APP and APLP2 die shortly after birth [27], [28]. Knock-in mice have been generated to identify the domain of APP responsible for these effects. Mutation of the first tyrosine residue, Tyr-682, of the YENPTY motif abolished Fe65 binding, and thus nuclear signaling by AICD [55]. APP Y682G knock-in mice crossed to an APLP2 knockout strain were found to be postnatally lethal with neuromuscular synaptic defects, resembling APP/APLP2 double knockouts [56]. This points towards a requirement for AICD/AL2ICD-mediated nuclear signaling in development, a function that cannot be performed by APLP1. This is further supported by the fact that knock-in of the sAPPβ or sAPPα ectodomains alone is not sufficient to rescue the synaptic deficits in APP/APLP2 knockout mice [57], [58]. Although the phenotypes in these mouse models could also originate from other aspects of APP biology–the Y682G knock-in has a dramatically increased production of sAPPα and the sAPP knock-ins also lack the Aβ sequence in addition to AICD–the current data combined with our experimental evidence strongly support a role of nuclear signaling by AICD and AL2ICD in synapse formation that cannot be performed by the AL1ICD.

We propose that AL1ICD does not have a direct physiological role in transcriptional regulation, but through sequestration of Fe65 by APLP1 this family member might act as a repressor of AICD-mediated nuclear signaling. We conclude that nuclear signaling is a prime function of the APP and APLP2 ICDs. Our results also caution the use of drugs inhibiting APP processing as treatment for AD because of possible interference with APP/APLP transcriptional function.

## Materials and Methods

### Ethics Statement

All animal experiments were performed according to the Swiss animal protection law (TschG) and approved by the local animal committee (Kantonales Veterinäramt Zürich; license 38/2011).

### Expression Constructs

APP-Citrine, HA-Fe65, and CFP-Tip60 expressing constructs have been described previously [4]. Clones of APLP1 (RZPD, Heidelberg, Germany) and APLP2 (courtesy of Stefan Kins, TU Kaiserslautern, Germany) were used to amplify open reading frames and ICDs and cloned into the pUKBK expression vector system [59] with either CMV or GAPDH promoters. Proteins were C-terminally tagged with Citrine (Cit), Cerulean (Cer), or streptavidin-binding protein (SBP), generating APP-Cer, APLP1-Cit, APLP1-Cer, APLP2-Cit, APLP2-Cer, and APP-SBP. For the constructs Cit-AICD, Cit-AL1ICD, Cit-AL2ICD, and Cer-AL2ICD fluorescence proteins were fused to the N-terminus of the respective ICD sequence. To generate Myc-Tip60, CFP was exchanged with a myc tag. GAPDH promoter driven 3myc-APP-3HA plasmid was constructed by inserting a 3myc-tag coding oligonucleotide after the APP signal peptide (SP) by restriction based cloning. For the corresponding APLP constructs, APP was replaced by open reading frames of APLP1 and APLP2. APP residues were changed to APLP1 residues by site-directed mutagenesis. Chimeric APP/APLP1 constructs, APP-AL1ICD and APLP1-AICD were derived from APP and APLP1 constructs by PCR-driven overlap extension as described [60].

### Cell Culture & Reagents

Human embryonic kidney cells (HEK-293, HEK) and mouse neuroblastoma N2a (N2a) were purchased from DSMZ (Braunschweig, Germany) and cultured at 37°C and 5% CO_2_ in Dulbecco’s modified eagle medium (DMEM; Invitrogen, Carlsbad, CA, USA), supplemented with 10% fetal calf serum (FCS) and 1% Penicillin/Streptomycin (Invitrogen). For transfection Lipofectamine 2000 (Invitrogen) was used according to the manufacturer’s protocol. Medium was replaced after three hours and cells fixed or homogenized 20–24 h after transfection if not indicated differently. To inhibit protein synthesis, cells were treated with 100 µg/ml cycloheximide (CHX; Sigma-Aldrich, St. Louis, MO, USA). Epoxomicin (EMD Millipore, Billerica, MA, USA) or MG-132 (Enzo Life Sciences, Farmingdale, NY, USA) were used to inhibit the proteasome, and DAPT (1 mM; Sigma-Aldrich) was used to inhibit γ-secretase. Primary cultures were prepared from cortices and hippocampi of 1-day-old C57Bl/6 mice as described [44].

### Immunocytochemistry

Cells were fixed for 20 minutes with 4% paraformaldehyde (PFA), washed with TBS containing 0.05% Triton X-100 and blocked for one hour with TBS containing 0.2% Triton X-100, 5% horse serum, and 5% goat serum. Fixed cells were incubated overnight with mouse anti-myc (Roche, Basel, Switzerland) or rat anti-HA (Roche) antibodies diluted 1∶100 in blocking solution. After washing, cells were incubated with Cy2, Cy3 or Cy5-conjugated secondary antibodies (1∶250; Jackson Laboratory, Ben Harbor, ME, USA) and embedded in Mowiol (Sigma-Aldrich).

### Live Antibody Incubation

HEK cells were, 24 hours after transfection, incubated with mouse anti-myc antibodies (1∶100; Roche) at 4°C for 10 or 30 minutes. After washing with ice-cold PBS, cells were fixed and stained for HA and myc tags.

### Western Blotting

Cell were lysed in a buffer, containing 150 mM NaCl, 10 mM Tris pH 7.6, 1% Triton X100, 0.25% NP40, and protease inhibitor (Roche) and centrifuged for 5 minutes at 14000 rpm [61]. Equal amounts of supernatants were separated on 10–20% Tricine gels (Invitrogen). Protein bands were visualized by ECL (Thermo Scientific, Rockford, IL, USA) using a Fuji LAS 3000 Imager. The following primary antibodies were used in this study: Anti-HA antibody (1∶1000; Roche), anti-GFP (1∶1000; Roche), anti-α-tubulin(1∶1000; Sigma-Aldrich), anti-GAPDH (1∶2000; Meridian Life Science, Memphis, TN, USA), anti-APP (1∶1000; Epitomics, Burlingame, CA, USA). Anti-mouse-HRP (1∶2000; Jackson Laboratory), anti-rat-HRP (1∶2000; Jackson Laboratory), and anti-rabbit-HRP (1∶2000; Jackson Laboratory) were used as secondary antibodies.

### Dynabeads-Streptavidin Pull-down

Pull-down of proteins fused to streptavidin-binding protein (SBP) was done as described previously [59]. Cells, transfected with APP-SBP and HA-Fe65, were lysed in homogenization buffer (150 mM KCl, 20 mM HEPES pH7.2, 10 mM NaCl, 5% Glycerol, 1% Triton X-100, 2 mM DTT, 5 mM phenanthroline, and protease inhibitor) and centrifuged for 10 minutes at 800 rpm. Supernatant was incubated with Dynabeads-Streptavidin M280 (Invitrogen) for 4 hours at 4°C. Beads were separated with a magnet and washed four times with homogenization buffer. For protein elution, beads were incubated with a buffer containing 700 nmol biotin for 30 minutes at 4°C. Equal amounts of protein elution were loaded on 10–20% Tricine gels.

### Confocal Microscopy & FRET Measurements

Images were acquired on a Leica TCS/SP2 confocal microscope (Leica, Wetzlar, Germany) and FRET measurements performed as described previously [4].

## Supporting Information

Figure S1 **ICDs derived from APP and APLP2, but not APLP1, form nuclear AFT complexes in HEK cells.** Confocal fluorescence images of HEK cells transfected with HA-Fe65, CFP-Tip60, and APP-Cit (row 1–2), HA-Fe65, CFP-Tip60, and APLP1-Cit (row 3–4), HA-Fe65, CFP-Tip60, and APLP2-Cit (row 5–6). AFT complex formation was observed in cells transfected with APP-Cit or APLP2-Cit. In contrast cells transfected with APLP1-Cit did not show AFT complex formation. Scale bar represents 13 µm.
(TIF)

Figure S2 **ICDs derived from APP and APLP2, but not APLP1, form nuclear AFT complexes in N2a cells.** Confocal fluorescence images of N2a cells transfected with HA-Fe65, CFP-Tip60, and APP-Cit (row 1–2), HA-Fe65, CFP-Tip60, and APLP1-Cit (row 3–4), HA-Fe65, CFP-Tip60, and APLP2-Cit (row 5–6). AFT complex formation was observed in cells transfected with APP-Cit or APLP2-Cit. In contrast cells transfected with APLP1-Cit did not show AFT complex formation. Scale bar represents 13 µm.
(TIF)

Figure S3 **APP family members show different subcellular localization and heterodimerization.** (A) Confocal fluorescence images of HEK cells transfected with APLP1-Cer and APP-Cit. Top row shows maximum projection and middle row single sections at different z positions**.** Note the intracellular localization of APP and the prominent localization of APLP1 at the plasma membrane. In contrast, the coexpression of APP and APLP2 shows a clear overlap and localization to the same intracellular compartments (bottom row). (B) Confocal fluorescence and FRET analysis of primary astrocytes expressing APP family members. APP-Cit was coexpressed with APP-Cer (top row), APLP1-Cer (second row), APLP2-Cer (bottom row). (C) Confocal fluorescence pictures and FRET analysis of HEK cells expressing APLP1-Cer and APP-Cit (top row), APLP2-Cer and APP-Cit (bottom row). (D) Confocal fluorescence pictures and FRET analysis of primary neurons expressing APP-Cer and APP-Cit (top row) and APLP1-Cer and APP-Cit (bottom row). In different cell types (B–D) coexpression of APP-Cit and APP-Cer revealed a strong FRET signal due to the presence of APP homodimers. Similarly, coexpression of APP-Cit and APLP2-Cer generated a FRET signal. In contrast, expression of APLP1-Cer and APP-Cit resulted in minimal FRET signal, indicating the near absence of APP/APLP1 heterodimerization. Scale bars represent 13 µm.
(TIF)

Figure S4 **Schematic presentation of APP family ICD sequences and APP mutations used in this study.**
(TIF)

Figure S5 **Replacement of most AICD residues by the corresponding AL1ICD residues does not ablate nuclear signaling.** Confocal fluorescence images of HEK cells cotransfected with HA-Fe65, CFP-Tip60 and the indicated APP-Cit mutants. Scale bar represents 13 µm.
(TIF)
