# Supplementary figures and images for: Turnover of Amyloid Precursor Protein Family Members Determines Their Nuclear Signaling Capability
Source: PLoS One. 2013 Jul 18;8(7):e69363. doi: 10.1371/journal.pone.0069363 (PMC3715505; doi:10.1371/journal.pone.0069363)

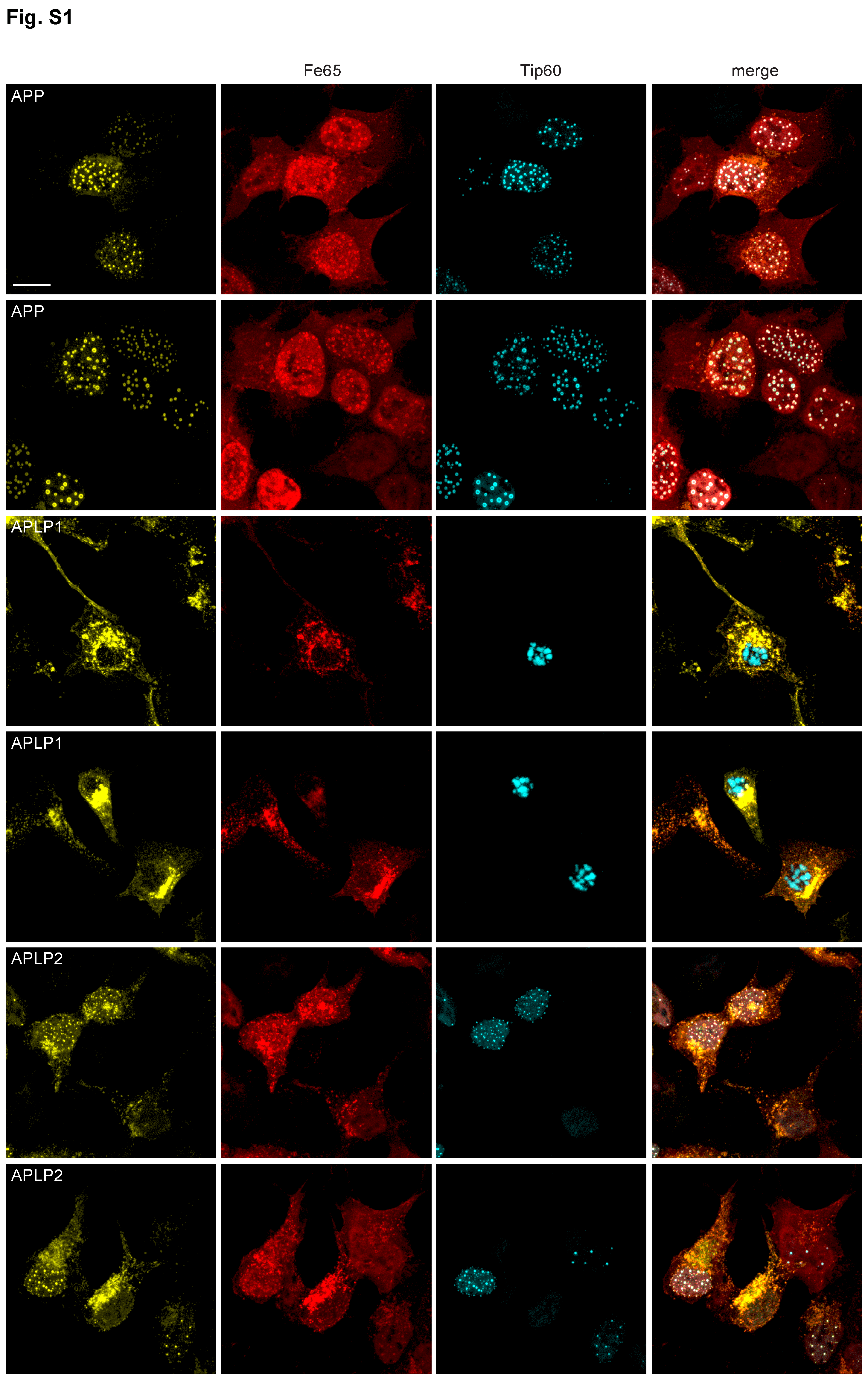

Supplement: Figure S1 — ICDs derived from APP and APLP2, but not APLP1, form nuclear AFT complexes in HEK cells. Confocal fluorescence images of HEK cells transfected with HA-Fe65, CFP-Tip60, and APP-Cit (row 1–2), HA-Fe65, CFP-Tip60, and APLP1-Cit (row 3–4), HA-Fe65, CFP-Tip60, and APLP2-Cit (row 5–6). AFT complex formation was observed in cells transfected with APP-Cit or APLP2-Cit. In contrast cells transfected with APLP1-Cit did not show AFT complex formation. Scale bar represents 13 µm. (TIF) [file pone.0069363.s001.tif]

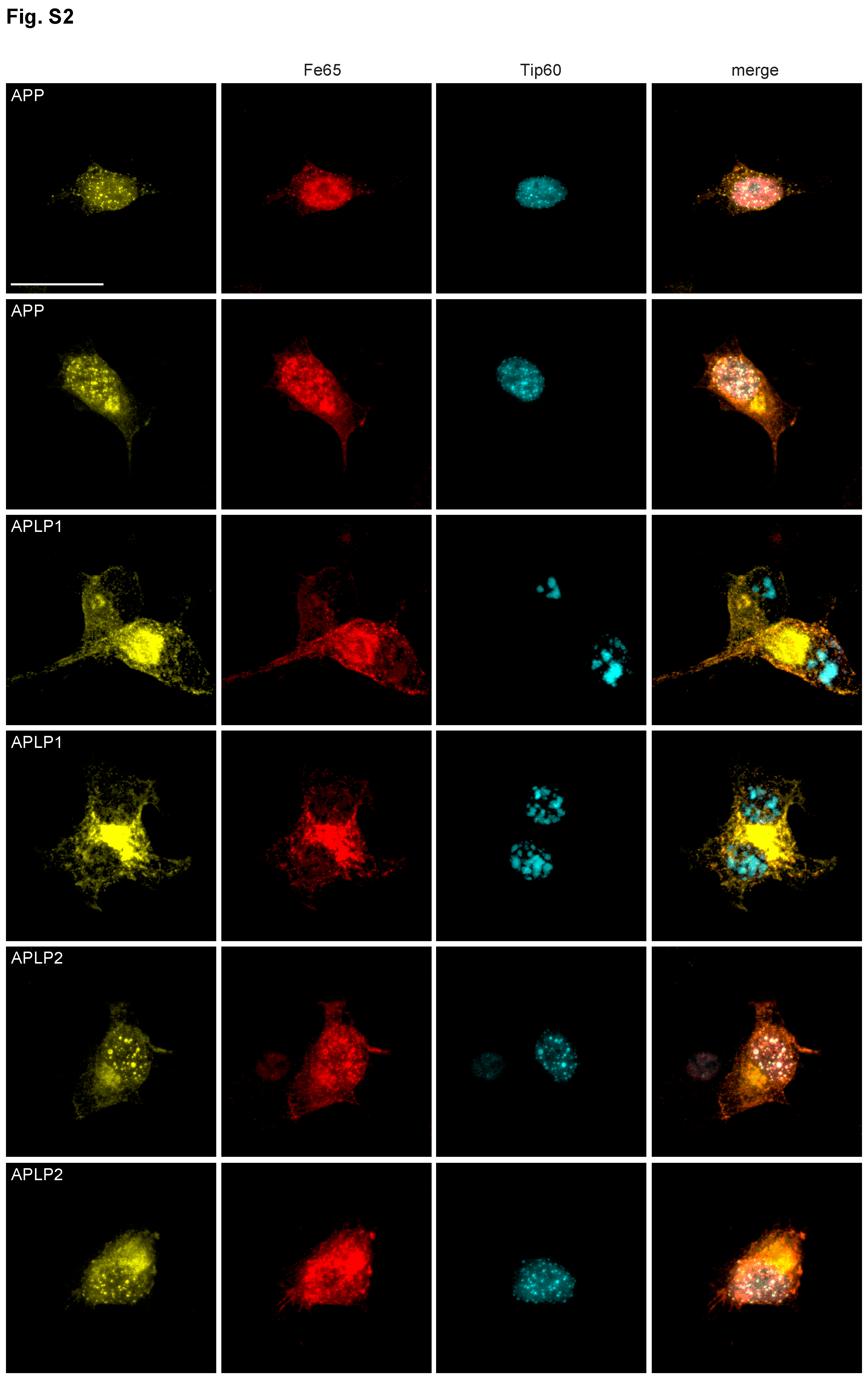

Supplement: Figure S2 — ICDs derived from APP and APLP2, but not APLP1, form nuclear AFT complexes in N2a cells. Confocal fluorescence images of N2a cells transfected with HA-Fe65, CFP-Tip60, and APP-Cit (row 1–2), HA-Fe65, CFP-Tip60, and APLP1-Cit (row 3–4), HA-Fe65, CFP-Tip60, and APLP2-Cit (row 5–6). AFT complex formation was observed in cells transfected with APP-Cit or APLP2-Cit. In contrast cells transfected with APLP1-Cit did not show AFT complex formation. Scale bar represents 13 µm. (TIF) [file pone.0069363.s002.tif]

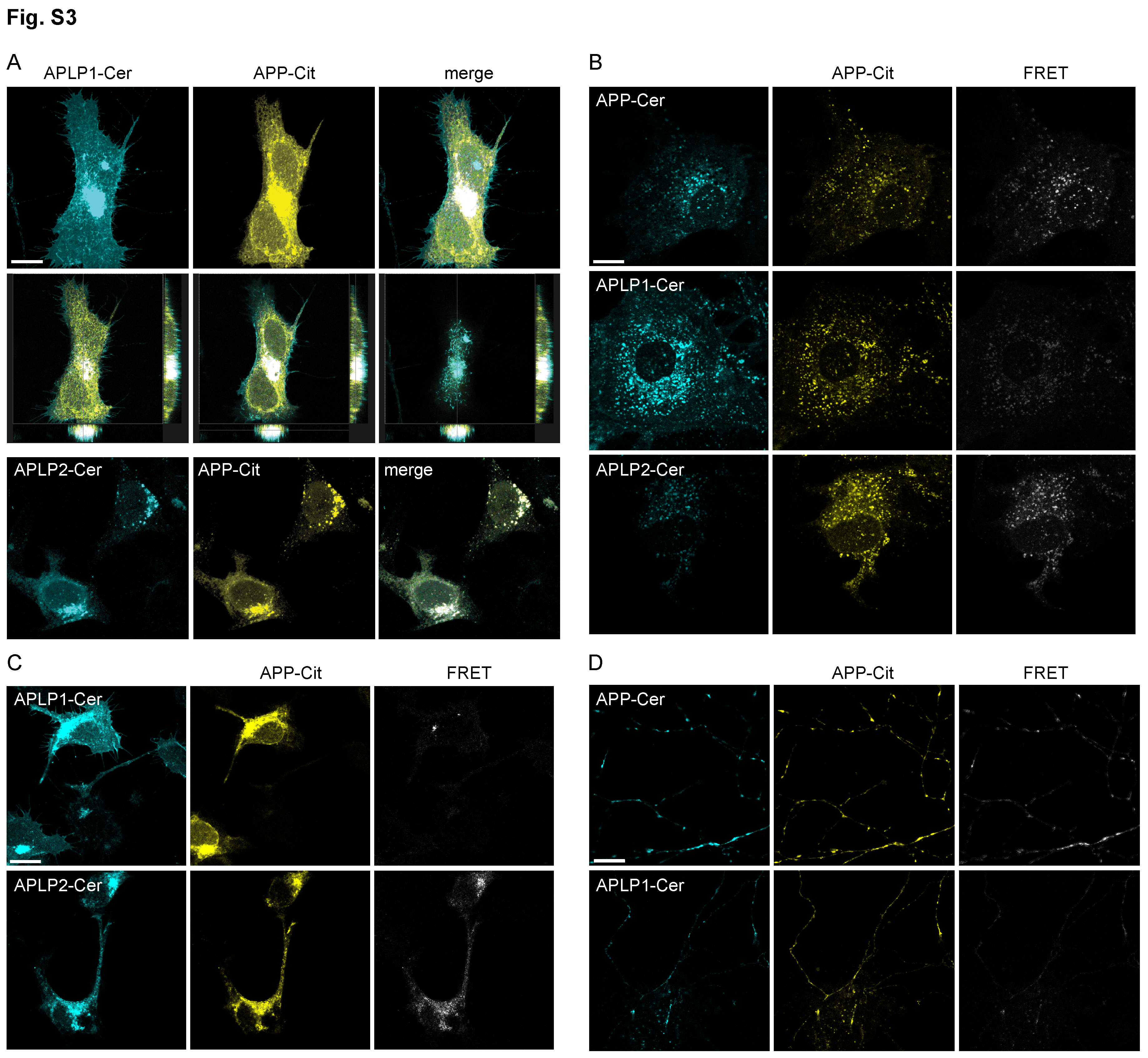

Supplement: Figure S3 — APP family members show different subcellular localization and heterodimerization. (A) Confocal fluorescence images of HEK cells transfected with APLP1-Cer and APP-Cit. Top row shows maximum projection and middle row single sections at different z positions. Note the intracellular localization of APP and the prominent localization of APLP1 at the plasma membrane. In contrast, the coexpression of APP and APLP2 shows a clear overlap and localization to the same intracellular compartments (bottom row). (B) Confocal fluorescence and FRET analysis of primary astrocytes expressing APP family members. APP-Cit was coexpressed with APP-Cer (top row), APLP1-Cer (second row), APLP2-Cer (bottom row). (C) Confocal fluorescence pictures and FRET analysis of HEK cells expressing APLP1-Cer and APP-Cit (top row), APLP2-Cer and APP-Cit (bottom row). (D) Confocal fluorescence pictures and FRET analysis of primary neurons expressing APP-Cer and APP-Cit (top row) and APLP1-Cer and APP-Cit (bottom row). In different cell types (B–D) coexpression of APP-Cit and APP-Cer revealed a strong FRET signal due to the presence of APP homodimers. Similarly, coexpression of APP-Cit and APLP2-Cer generated a FRET signal. In contrast, expression of APLP1-Cer and APP-Cit resulted in minimal FRET signal, indicating the near absence of APP/APLP1 heterodimerization. Scale bars represent 13 µm. (TIF) [file pone.0069363.s003.tif]

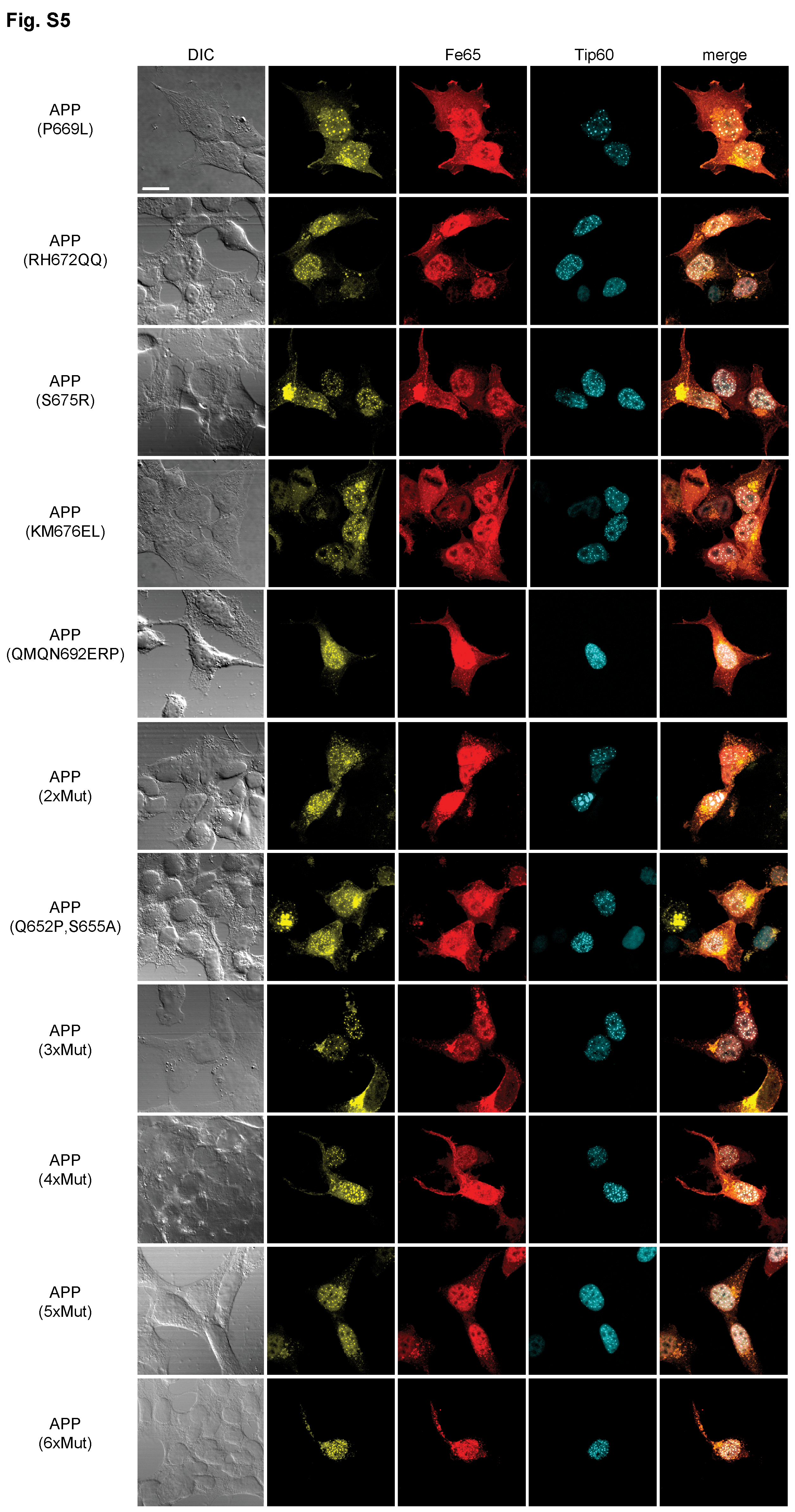

Supplement: Figure S5 — Replacement of most AICD residues by the corresponding AL1ICD residues does not ablate nuclear signaling. Confocal fluorescence images of HEK cells cotransfected with HA-Fe65, CFP-Tip60 and the indicated APP-Cit mutants. Scale bar represents 13 µm. (TIF) [file pone.0069363.s005.tif]
